# Supplementary material for: Circuit dynamics of binocular conflict in mouse primary visual cortex
Source: Front Syst Neurosci. 2026 May 29;20:1786396. doi: 10.3389/fnsys.2026.1786396 (PMC13260517; doi:10.3389/fnsys.2026.1786396)
Supplement: Supplementary file 1 [file Supplementary_file_1.pdf]

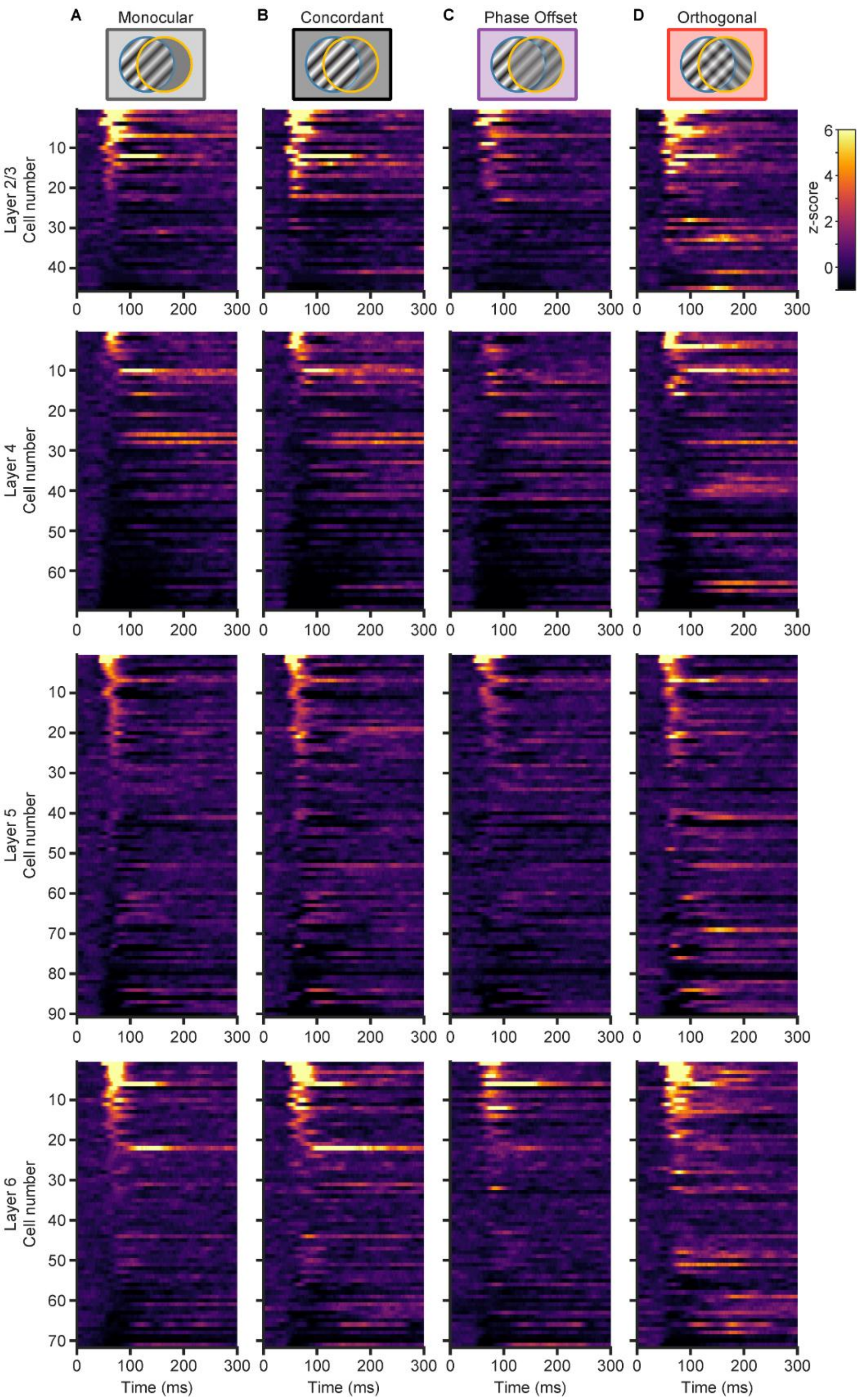

Figure S1. Regular-spiking single unit responses to different stimulus conditions. Z-scored raster plots of regular-spiking (RS) single units for Monocular **(A)**, Concordant **(B)**, Phase Offset **(C)**, and Orthogonal **(D)** stimuli. Units were assigned to L2/3, L4, L5, or L6 based on the location of the electrode contact with the maximal single unit waveform, then sorted based on their average activity between 40-80ms in the Monocular condition. Differences between conditions and layers are quantified in **Figure 5**.

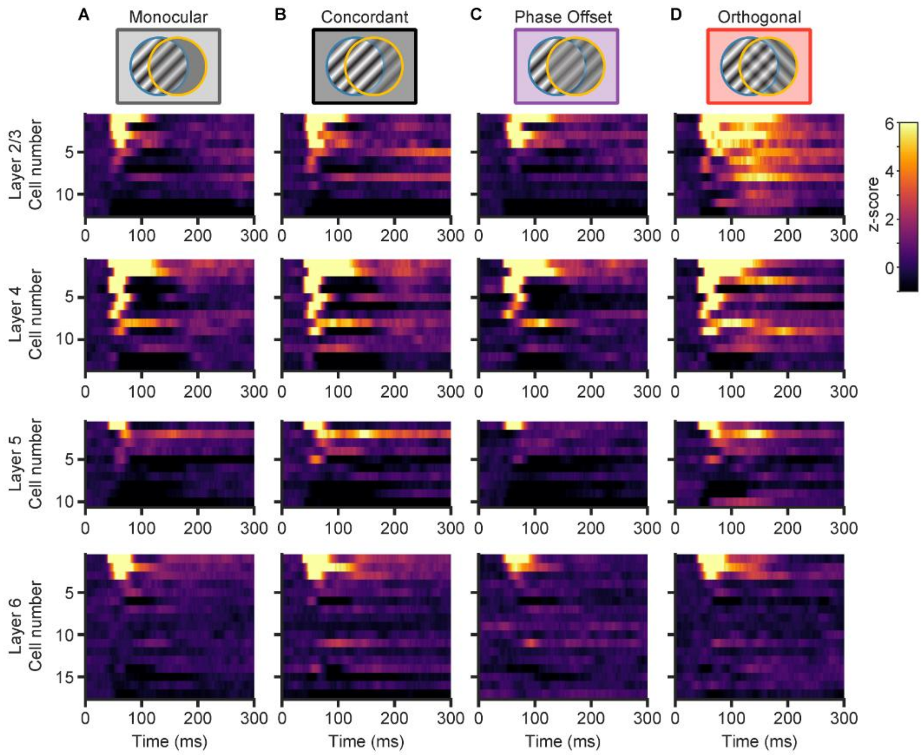

Figure S2. Fast-spiking single unit responses to different stimulus conditions. Z-scored raster plots of fast-spiking (FS) single units for Monocular **(A)**, Concordant **(B)**, Phase Offset **(C)**, and Orthogonal **(D)** stimuli. Units were assigned to L2/3, L4, L5, or L6 based on the location of the electrode contact with the maximal single unit waveform, then sorted based on their average activity between 40-80ms in the Monocular condition. Differences between conditions and layers are quantified in **Figure 5**.

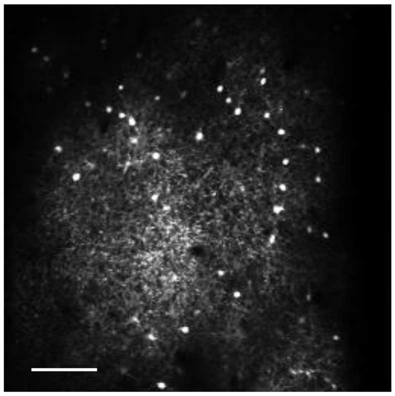

Figure S3. Calcium imaging of SOM+ neurons. Example FOV for a mouse expressing GCaMP6f in somatostatin-positive (SOM+) cells imaged 200µm below pia (L2/3). White circles represent individual neurons. Scale bar=100µm.
